# Role of Serum Biomarkers in Differentiating Periprosthetic Joint Infections from Aseptic Failures after Total Hip Arthroplasties

**DOI:** 10.3390/jcm13195716

**Published:** 2024-09-25

**Authors:** Flaviu Moldovan

**Affiliations:** Orthopedics—Traumatology Department, Faculty of Medicine, George Emil Palade University of Medicine, Pharmacy, Science, and Technology of Targu Mures, 540142 Targu Mures, Romania; flaviu.moldovan@umfst.ro; Tel.: +40-754-671-886

**Keywords:** periprosthetic joint infection, serum biomarkers, total hip arthroplasty, diagnostic performance

## Abstract

**Background/Objectives**: Periprosthetic joint infection (PJI) is a disastrous complication after joint replacement procedures as the diagnosis remains a significant challenge. The objective of this study is to assess the accuracy and test the interdependency of the proposed compound serum biomarkers for the diagnosis of PJI after total hip arthroplasties (THA). **Methods**: From January 2019 to December 2023, 77 consecutive cases that underwent revision total hip arthroplasties (rTHA) were included in a single−retrospective, observational cohort study. A total of 32 arthroplasties were classified as having septic complications using the European Bone and Joint Infection Society (EBJIS) definition from 2021, while the other 45 cases were assigned as aseptic failures (AF). **Results**: In the univariate analysis between the two groups created, statistically significant differences (*p* < 0.005) were found for the following variables: time from primary arthroplasty to symptom onset (Time PA−SO), neutrophil count, Lymphocyte count, haematocrit level (HCT) and haemoglobin level (HGB), C−reactive protein (CRP), the neutrophil lymphocyte ratio (NLR), platelet lymphocyte ratio (PLR), monocyte lymphocyte ratio (MLR), systemic inflammation index (SII), systemic inflammation response index (SIRI), and aggregate inflammation systemic index (AISI). The ROC curve analysis showed that the SII (sensitivity 90.6% and specificity 62.2%) and the NLR (sensitivity 84.4% and specificity 64.4%) are the most accurate biomarkers. The multivariate analysis confirmed that NLR > 2.63 (*p* = 0.006), PLR > 147 (*p* = 0.021), MLR > 0.31 (*p* = 0.028), SII > 605.31 (*p* = 0.002), SIRI > 83.34 (*p* = 0.024), and AISI > 834.86 (*p* = 0.011) are all closely related to PJI diagnosis independently. **Conclusions**: The proposed serum biomarkers can be correlated with PJI diagnosis with the reserve of relatively low specificities.

## 1. Introduction

Periprosthetic joint infection (PJI) constitutes a particular type of infection associated with joint replacement procedures, as well as a devastating complication with a substantial economic burden, and high morbidity and mortality rates [1]. It is estimated that around 12–15% of total hip arthroplasty revisions are performed due to this complication [2]. Changes in the parameters of prosthetic cement can affect the aseptic condition of prostheses [3]. Key factors include the cement’s setting time, strength, biocompatibility, and antibacterial properties. Faster-setting cement reduces the risk of contamination, while materials with greater strength and antibacterial properties promote hygiene. A smooth, non-porous surface makes the prosthesis easier to clean, reducing the risk of infections [4,5].

Precise diagnosis of PJI can assure prompt management with successful outcomes, although it remains a current challenge. This is due especially to low-virulent organisms that form a biofilm matrix around the implant [6]. Stepwise diagnostic algorithms were recently proposed, as they include a mixture consisting of medical characteristics, serum, and synovial analysis, together with histologic and microbiologic examinations [7]. Among them, serum inflammatory markers were proven to be a non-invasive, rapid, and cheap way to aid diagnosis in the preoperative period. However, their efficiency remains questionable [8]. A recent study by Sun et al. [9] concluded that serum procalcitonin alone is not a reliable parameter, but, in association with C−reactive protein (CRP), erythrocyte sedimentation rate, serum platelet, and fibrinogen, can increase the diagnostic accuracy. Other studies [10,11], reached similar results by examining other serum markers like interleukin-6 (IL−6), tumor necrosis factor alfa (TNF−α), and D−dimer levels. As for synovial fluid parameters, the most reliable are the leucocyte esterase, detected by a urine dipstick test and α-defensin, an antimicrobial peptide which is secreted by neutrophils in response to different pathogens [12]. PJI definitions have suffered several changes in the past years; the most notable ones are the Musculoskeletal Infection Society (MSIS, from 2011), the Infectious Diseases Society of America (IDSA, from 2012), and International Conference on Musculoskeletal Infection (ICM, from 2013 which was redefined in 2018). All these definitions had an issue of presenting a binary outcome (with or without infection) by using tests that were neither 100% specific nor sensitive. The most recent definition given by European Bone and Joint Infection Society (EBJIS) in 2021 [13] is supported by previous ones, such as the MSIS, but also by the European Society of Clinical Microbiology and Infectious Diseases (ESCMID) and the Study Group for Implant-Associated Infections (ESGIAI). This new definition proposed three groups (‘infection unlikely’, ‘likely’ and ‘confirmed’) and has retained only CRP as serum parameter, making it an ‘infection likely’ criteria. Special considerations were given to the fact that some tests have a very high sensitivities with low specificities (e.g., nuclear imaging) and some highly specific findings (e.g., sinus formation, identification of two positive samples that have the same microorganism) that are not present in the most periprosthetic infections reported. Thus, identifying novel and accurate serum biomarkers for early PJI diagnosis represents a wide area of research.

It was proven that serum white blood count (WBC) has limited role for routine check-up of PJI due to low sensitivities (55%) and specificities (66%), although many clinicians still use it due to the ease of availability [14]. Serum neutrophil count is well known to increase in many infectious pathologies [15], which makes it a reference parameter for PJI identification when used in conjunction with other diagnostic tools. Zareifar et al. [16] demonstrated that with an increase in platelet count (PLT) there is a decrease in the average volume of platelets (MPV) in active infectious diseases in comparison to recovered patients. Using this information, the PLT/MPV ratio was demonstrated to be significantly increased in PJI groups [17]. Recruitment of bloodstream monocytes poses a significant role in controlling and clearing of fungal, bacterial, viral, and protozoal infections [18]. Moreover, it was demonstrated that persistent lymphopenia four days after diagnosing sepsis can predict premature and delayed mortality and can be used as a sepsis-induced immunosuppression biomarker [19].

These serum parameters have been explored in recent studies to compute different biomarkers that can quantify the inflammatory process and predict outcomes for different medical conditions. For example, serum neutrophil lymphocyte ratio (NLR), serum monocyte lymphocyte ratio (MLR), and serum platelet lymphocyte ratio (PLR) were used to differentiate between and predict outcomes for viral and bacterial pneumonia in intensive care units [20]. Combined blood indexes of systemic inflammation, such as serum systemic inflammation index (SII), serum systemic inflammation response index (SIRI), or serum aggregate inflammation systemic index (AISI), were used predict the severity and survival rate of COVID−19 or pneumoconiosis patients [21,22]. In the field of Orthopedics and Traumatology, these biomarkers have been useful to quantify the magnitude of surgical trauma and set recommendations for different osteosynthesis protocols [23,24].

To date, a few studies have investigated and correlated this type of serum biomarker with periprosthetic infections, but without reaching a clear and general accepted consensus. This study’s hypothesis is that compound blood-derived indexes can be an adjuvant diagnostic tool for the diagnosis of PJI when used in conjunction with other well-established parameters. Hence, the objective of this research is to estimate the precision of serum NLR, serum PLR, serum MLR, serum SII, serum SIRI, and serum AISI in diagnosing PJI after total hip arthroplasties and to test their interdependency in differentiating this dreadful complication from aseptic failures (AF).

## 2. Materials and Methods

### 2.1. Study Design and Patient Enrollment

**Figure 1:**
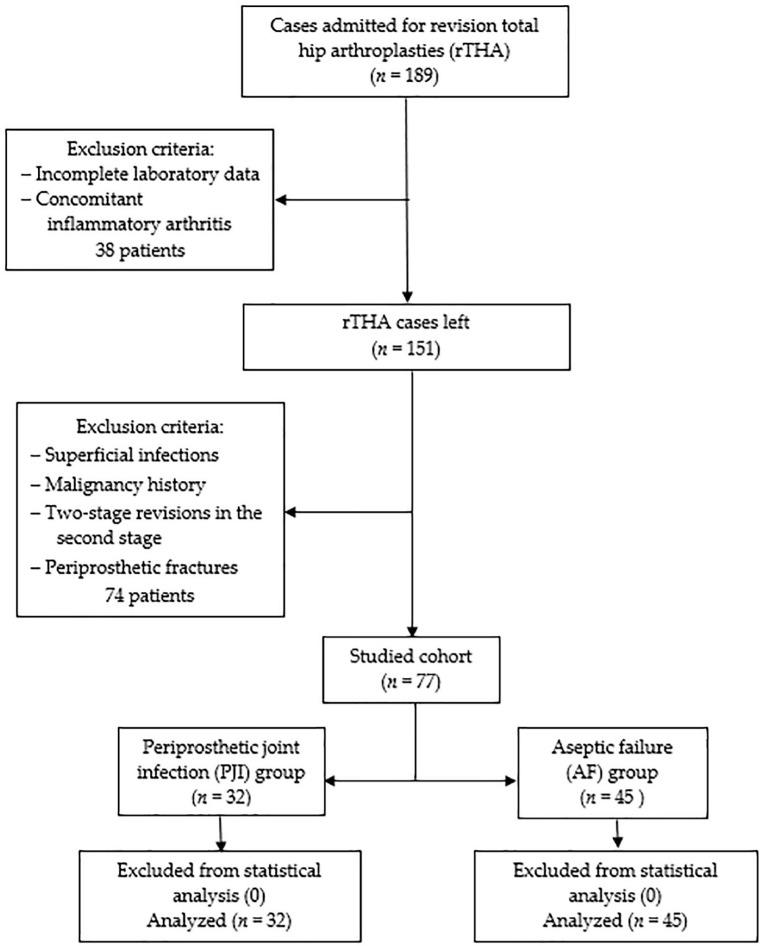
The studied cohort flowchart diagram.

Following Institutional Review Board approval, a retrospective single-center cohort study was conducted according to the Helsinki Declaration. The statistical protocol followed was the extraction of the representative population using the inclusion/exclusion criteria, forming the two samples according to the selected PJI definition, descriptive statistics, and inferential statistics. Initially, all patients admitted with revision total hip arthroplasties (rTHA) between January 2019 and December 2023 at the Department of Orthopedics—Traumatology from Targu Mures County Emergency Hospital in Romania were included. Among clinical reasons for admission were pain, reduced range of movement, swelling, fever, erythema, wound drainage, and sinus formation. Exclusion criteria consisted of incomplete laboratory data, concomitant inflammatory arthritis (e.g., Rheumatoid arthritis, Ankylosing spondylitis), superficial infections, malignancy history, two-stage revisions in the second stage, and periprosthetic fractures. Patients with these conditions were removed from the statistical analysis to reduce the bias associated with an already increased inflammatory status and intricate source of microorganisms. The EBJIS (European Bone and Joint Infection Society) definition from 2021 [13] was used to from two groups: the periprosthetic joint infection group (PJI group, *n* = 32) and aseptic failure (AF group, *n* = 45). According to these criteria, ‘likely’ infections were included in the AF group with an assigned conscientious follow-up. During revision surgery, a minimum of three solid tissue samples were collected for tissue culture and histopathological examination. Incubation for microbiological cultures was at least 14 days, as the protocol recommends. None of the 77 cases were removed from statistical analysis as no sampling, measurement, or processing errors could be identified. In Figure 1, a detailed flowchart diagram of the studied cohort is presented.

### 2.2. Acquisition of Data

Variables extracted from the department’s digital database that were used for statistical analysis were: (1) demographic details: age, sex, alcohol consumption, tobacco usage, obesity (body mass index ≥ 25), living area; (2) associated comorbidities: hypertension, ischemic cardiomyopathy (IC), atrial fibrillation (Afib), diabetes mellitus (DM), chronic kidney disease (CKD), dyslipidemia, chronic venous insufficiency (CVI) below stage 6 (active ulcer); (3) time related factors: time from primary arthroplasty to symptom onset (Time PA−SO), length of hospital stay (LOS); (4) preoperative laboratory analysis: neutrophilcalculation, lymphocytecalculation, monocytecalculation, platelet (PLT) calculation, glucose, activated partial thromboplastin clotting time (APTT), International normalized ratio (INR), hematocrit (HCT), hemoglobin levels (HGB), C−reactive protein (CRP).

### 2.3. Determination of Serum Biomarkers

The day before revision surgery, routine cubital fasting venous blood samples were collected and tested within two hours by the hospital’s laboratory. The results were used to determine the following biomarkers of inflammation: serum neutrophil lymphocyte ratio (NLR)—the division of total neutrophils and total lymphocytes; serum platelet lymphocyte ratio (PLR)—the division of total platelets and total lymphocytes; serum monocyte lymphocyte ratio (MLR)—the division of total monocytes and total lymphocytes; serum systemic inflammation index (SII)—the multiplication of the total neutrophils with the total platelets divided by the total lymphocytes; serum systemic inflammation response index (SIRI)—the multiplication of total monocytes with the total platelets divided by the total lymphocytes; and serum aggregate inflammation systemic index (AISI)—the product between total neutrophiles, total monocytes, and total platelets divided by total lymphocytes.

### 2.4. Statistical Analysis

All statistical analysis was carried out using SPSS-IBM (SPSS, Inc., Chicago, IL, USA) for Widows, version 29.0.2. The assessment of the normal distribution of continuous variables was carried out using the Kolmogorov-Smirnov test, which ensured the correct analysis of this variables with either Student’s *t*-test as well as the Wilcoxon rank-sum test. As for categorical variables, intergroup significances were investigated with the Chi-square test or Fisher’s exact test according to the size of the attributed cells. A statistically significant value was considered when *p* < 0.05. To identify the diagnostic power (cut-off values, AUC—area below the curve, 95% CI—confidence intervals, sensitivity and specificity) of the proposed serum biomarkers, a receiver operating characteristic curve (ROC) was plotted. Youden’s index (Youden index = sensitivity + specificity − 1, range 0 to 1) was used to identify the optimal cut-off value for each of them. An acceptable multivariate logistic regression model (determined with the Hosmer-Lemeshow test with a *p* value higher than 0.05) was projected to determine the independency of the biomarkers. The strengths of the correlations were assessed by odds ratios (OR with a 95% CI).

## 3. Results

A total of 77 cases admitted for rTHA were included in the final analysis. According to EBJIS criteria, 32 cases (41.56%) were assigned to the PJI group, and 45 cases (58.44%) were assigned to the AF group. The mean age (*p* = 0.687) in the PJI group was 62 (17 males and 15 females) and the mean age in the AF group was 64 with (14 males and 21 females).

The proposed serum biomarkers (NLR, PLR, MLR, SII, SIRI, and AISI) were evaluated using ROC curve analysis, to identify the most optimal cut-off points in diagnosing infection (Table 1).

Abbreviations: NLR—serum neutrophil lymphocyte ratio; PLR—serum platelet lymphocyte ratio; MLR—serum monocyte lymphocyte ratio; SII—serum systemic inflammation index; SIRI—serum systemic inflammation response index; AISI—serum aggregate inflammation systemic index; ROC—receiver operating characteristic; AUC—area under the curve; CI—confidence interval; PPV—positive predictive value; NPV—negative predictive value. A *p* value of < 0.05 was accepted as being statistically significant (bold).

**Figure 2:**
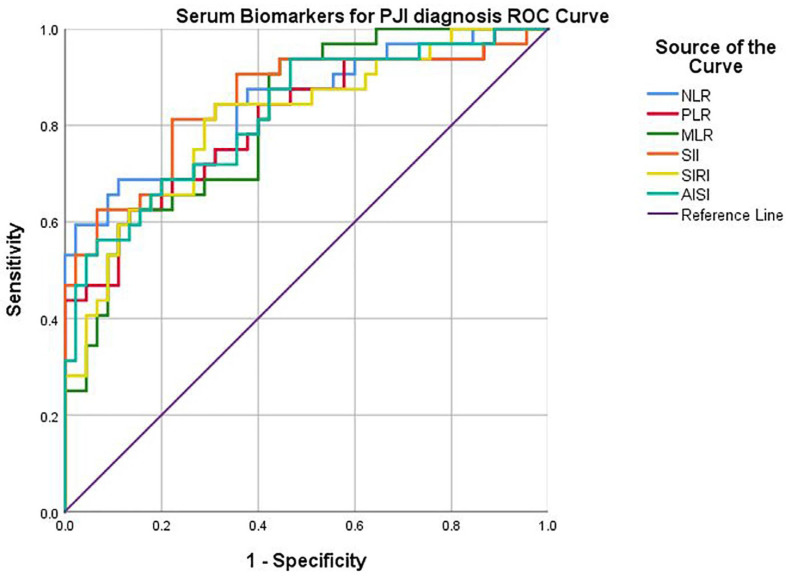
ROC curve of serum biomarkers for PJI diagnosis.

By plotting the curves (Figure 2), the corresponding AUC (area under the curve), sensitivity, and specificity were determined. A good diagnostic power was found for all biomarkers: NLR (cut-off 2.63, AUC 0.843, sensitivity 84.4%, specificity 64.4%, *p* < 0.0001), PLR (cut-off 147, AUC 0.806, sensitivity 75.0%, specificity 68.9%, *p* < 0.0001), postoperative MLR (cut-off 0.31, AUC 0.813, sensitivity 81.3%, specificity 60.0%, *p* < 0.0001), SII (cut-off 605.31, AUC 0.851, sensitivity 90.6%, specificity 62.2%, *p* < 0.0001), SIRI (cut-off 83.34, AUC 0.810, sensitivity 78.1%, specificity 71.1%, *p* < 0.0001), AISI (cut-off 834.86, AUC 0.822, sensitivity 78.1%, specificity 64.4%, *p* < 0.0001).

Statistically significant differences between the PJI group and the AF group (Table 2) were identified for the following variables: time from primary arthroplasty to symptom onset (*p* < 0.0001), neutrophil count (*p* = 0.022), lymphocyte count (*p* < 0.0001), HCT level (*p* = 0.004), HGB level (*p* = 0.014), and CRP level (*p* = 0.006).

**Figure 3:**
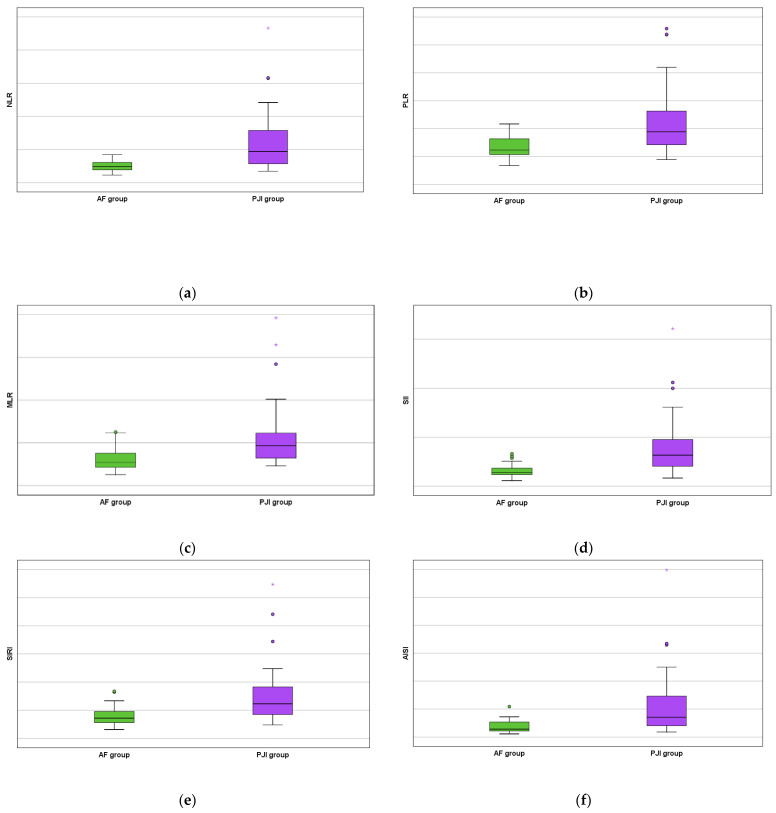
AF versus PJI groups boxplots concerning the biomarkers: (**a**) serum neutrophil lymphocyte ratio; (**b**) serum platelet lymphocyte ratio; (**c**) serum monocyte lymphocyte ratio; (**d**) serum systemic inflammation index; (**e**) serum systemic inflammation response index; and (**f**) serum aggregate inflammation systemic index. The mild outliers are represented by circles and the extreme outliers are represented by asterisks.

Moreover, all studied serum biomarkers showed a high statistical value (NLR with *p* < 0.0001 by using the Fisher exact test, PLR with *p* < 0.0001 by using the Chi-square test, MLR with *p* = 0.001 by using the Chi-square test, SII with *p* < 0.0001 by using the Fisher exact test, SIRI with *p* < 0.0001 by using the Chi-square test, and AISI with *p* < 0.0001 by using the Fisher exact test) as increased values were obtained for the PJI group (Figure 3).

An overview of all detected microorganisms is presented in Table 3. Methicillin-susceptible *Staphylococcus aureus* was the most frequently isolated (*n* = 10, 31.25%), followed by Methicillin-resistant *Staphylococcus aureus* (*n* = 7, 21.88%), Streptococci (*n* = 3, 9.38%), and Pseudomonas aeruginosa (*n* = 3, 9.38%).

These microorganisms were further subdivided into two groups: the Methicillin-resistant group and the Methicillin-susceptible *Staphylococcus aureus* group, as these were pre-dominant and the other types of pathogens identified (Table 4). There were no statistically significant differences in the tested serum biomarkers (NLR, PLR, MLR, SII, SIRI, and AISI) between the two groups (*p* > 0.05), which suggests that they cannot be of use in differing the species of pathogen.

In the final step of the research, a multivariate logistic regression model that included the serum biomarkers was projected (Table 5). The goodness of fit was acceptable according to the by Hosmer—Lemeshow test: X^2^ = 8.066, *p* = 0.327 and Nagelkerke R^2^ = 0.530. The values obtained in the equation showed that periprostatic infection can be correlated independently with all the elements involved: NLR (OR 17.52, 95% CI 4.65–66.66, *p* = 0.006), PLR (OR 9.45, 95% CI 3.08–29.39, *p* = 0.021), MLR (OR 6.25, 95% CI 2.24–18.28, *p* = 0.028), SII (OR 34.22, 95% CI 14.18–279.92, *p* = 0.002), SIRI (OR 6.64, 95% CI 2.39–18.40, *p* = 0.024) and AISI (OR 10.66, 95% CI 3.56–31.95, *p* = 0.011).

## 4. Discussion

The lack of ‘gold standard’ tests make PJI diagnosis difficult, as well as to distinguish it from aseptic cases of loosening. This may pose clinical implications, as treatment management is challenging and differs substantially [25,26]. At the same time, it is also essential to have wide access to accurate, rapid, and easy to obtain diagnostic tools to minimize the negative impact of this complication. This research analyzes the potential in the management of THA infections by the proposed serum biomarkers as non-invasive parameters.

All of the serum biomarkers performed well with high AUCs (>0.800) and showed a significant association (*p* < 0.0001) with the infectious process. Among them, the serum SII > 605.31 (sensitivity 90.6% and specificity 62.2%), followed by serum NLR > 2.63 (sensitivity 84.4% and specificity 64.4%), are the most accurate. It can be deduced from the multivariate logistic regression model that the studied biomarkers can be correlated independently with the detection of periprosthetic infection. Among them, it is confirmed that SII (OR: 34.22) and NLR (OR: 17.52) are the most reliable. Nonetheless, in the univariate analysis, clear differences can be seen between the two groups in the neutrophil count (*p* = 0.022) and lymphocyte count (*p* < 0.001), which are major components of the two serum biomarkers. As Delano et al. [27] have demonstrated, infection states of the organism downregulation of chemokine CXCL12 occurs, which leads to mobilization of neutrophils from the bone marrow into the peripheral bloodstream. Moreover, one of the suppression characteristics sustained by the immune system in sepsis is lymphocyte count decreases starting from early phases, process that continues up to 28 days [28]. These findings are in accordance with the study conducted by Yu et al. [29], who suggest that the NLR index with a similar cut−off value of 2.13 is a more accurate than CRP for screening early PJIs. However, another study [30] demonstrated that the SII index is even more efficient (sensitivity 85.5% and specificity 71.2%) than the NLR and PLR in predicting sepsis mortality.

From the two groups, the HGB (*p* = 0.014) and HCT (*p* = 0.004) were seen to be statistically lower for the PJI group. This observation is in line with the results of Telang et al. [31], who identified that HGB levels of <12 g/dL (in the case of females) or <13 g/dL (in the case of males), abnormal platelet count, and elevated NLR, PLR, and SII are all risk factors for PJI development in obese patients. As expected, the C−reactive protein was also statistically different (*p* = 0.006) with mean values of 1.08 mg/dL, although the aseptic failure group presented minor elevations with mean values of 0.86 mg/dL. Interestingly, combined indexes using CRP as a component, such as the serum CRP-to-albumin ratio (CAR), were shown not to outperform this inflammatory parameter when used alone [32].

The MLR and SIRI have proven their utility in predicting successful prothesis reimplantation after two-stage revision surgeries (“1.5−stage exchange arthroplasties”) in chronic cases of PJI [33,34]. A well-defined sequence exists as neutrophils are the first to accumulate into inflammatory sites, ushering monocytes after them as neutrophil-derived cathelicidin promotes endothelial adhesion of the later cells [35]. This interdependency between white cells can explain why composite indexes that contain monocytes, such as MLR (OR: 6.25, *p* = 0.028), SIRI (OR: 6.64, *p* = 0.024), and AISI (OR: 10.66, *p* = 0.011) had lower affinity towards periprosthetic infections diagnosis. Platelets are recognized to have a multifaced response to pathogens and infections as they can modulate leukocyte behavior in terms of phagocytosis and induce effector functions, such as neutrophil extracellular traps (NETs) production [36]. Klemt et al. [37] demonstrated for the PLR index similar a sensitivity (75.92% vs. 75.0%) but slightly higher specificity (82.78% vs. 68.9%) compared to the present study in identifying PJI after THA.

Different time points have used in the scientific literature to classify PJIs, but the classification system proposed by Conventry where early-onset PJI is defined as under 3 months after initial surgery (initiated due to virulent microorganisms with origin at the operation site), delayed-onset PJI defined as after 3 months but under 12–24 months after surgery (initiated due to less virulent microorganisms with the same operation site origin) and late-onset PJI defined as more than 12–24 months after surgery (initiated due to very low-grade microorganisms through hematogenous infection), is generally accepted [38]. The mean time of symptom onset for the PJI group in this study was 381 days (approximately 12.5 months), which suggests that most of the studied cases were delayed-onset infections. In terms of frequency, Methicillin-susceptible *Staphylococcus aureus* (MSSA) with a portion of 31.25%, followed by Methicillin-resistant *Staphylococcus aureus* (MRSA) with a portion of 21.88%, were seen to be the leading pathogens. Infections associated with orthopedic implants were proven to be due to *Staphylococcus aureus*, epidermidis, and other coagulase−negative staphylococci in most cases (50%), as these pathogens can be involved in both early and delayed-onset cases [39,40].

In this study, a set of limitations were identified. Firstly, a heterogeneous population included in a small sample size was analyzed, which may have decreased the statistical power of the conclusions. But it must be considered that PJI is not a common condition, despite increasing numbers of patients affected. Secondly, the retrospective single-center design could have produced a selection bias affecting the results. This situation can be corrected by developing multicenter prospective research. Thirdly, some AF cases may have been diagnosed as PJI cases due to the reliability of the EBJIS definition, although studies [41,42] suggest that it does not over-diagnose infections when compared to the criteria proposed by the International Consensus Meeting (ICM from 2018) and the Infectious Diseases Society of America (IDSA from 2013). Lastly, the optimal threshold values of the proposed serum biomarkers in diagnosing PJI need to further be examined due to different detection tools used [43]. Overall, by overcoming these limitations the studied biomarkers could be integrated in current diagnostic protocols through a standardized approach as their superiority is demonstrated over the standard of care, the clinical pathway is clearly understood, and real-world performance is attributed.

## 5. Conclusions

A clear association between the proposed serum biomarkers and preoperative PJI diagnosis was established with the reserve relatively low specificities within a range of 60−70%. This can be attributed to the fact that low-grade infections that were also included in the study are known to produce a reduced inflammatory and immunologic response.

The data from this study suggest that compound biomarkers such as SII with cut-off values >605.31, as well as more commonly biomarkers such as NLR with cut-off values >2.63, may increase the accuracy of PJI diagnosis when used in conjunction with other well-established parameters. In this manner, the information related to inflammatory-infectious status of the patient can be better interpreted by the clinician, as it is easy to obtain, convenient, and non-invasive. However, further studies evaluating the diagnostic rate of these serum biomarkers on low-grade and late infections are still needed to validate the results. It is also important to acknowledge that their level can vary with diverse population demographic and lifestyle factors such as age and sex, thus future studies can additionally provide more precise data on this aspect.

## Figures and Tables

**Table 1:** Values of serum biomarkers in diagnosing infection identified using ROC curve analysis.

Variables	Cut-Off Values	AUC	95% CI	Sensitivity	Specificity	PPV	NPV	*p* Value
NLR	2.63	0.843	0.751–0.935	84.4%	64.4%	0.62	0.84	***p* < 0.0001**
PLR	147	0.806	0.705–0.907	75.0%	68.9%	0.63	0.79	***p* < 0.0001**
MLR	0.31	0.813	0.720–0.907	81.3%	60.0%	0.59	0.81	***p* < 0.0001**
SII	605.31	0.851	0.758–0.943	90.6%	62.2%	0.64	0.90	***p* < 0.0001**
SIRI	83.34	0.810	0.712–0.909	78.1%	71.1%	0.66	0.84	***p* < 0.0001**
AISI	834.86	0.822	0.726–0.917	78.1%	64.4%	0.93	0.71	***p* < 0.0001**

Abbreviations: NLR—serum neutrophil lymphocyte ratio; PLR—serum platelet lymphocyte ratio; MLR—serum monocyte lymphocyte ratio; SII—serum systemic inflammation index; SIRI—serum systemic inflammation response index; AISI—serum aggregate inflammation systemic index; ROC—receiver operating characteristic; AUC—area under the curve; CI—confidence interval; PPV—positive predictive value; NPV—negative predictive value. A *p* value of < 0.05 was accepted as being statistically significant (bold).

**Table 2:** Univariate analysis between PJI group and AF group.

Variables	All Patients (*n = 77*)	PJI Group (*n = 32*)	AF Group (*n = 45*)	*p* Value
**Demographic details**				
Age (years), mean ± SD	63.16 ± 12.74	61.91 ± 15.49	64.04 ± 10.45	0.687
Gender, *n* (%)				0.986
Male	41 (53.2)	17 (53.1)	24 (53.3)	
Female	36 (46.8)	15 (46.9)	21 (46.7)	
Alcohol (yes), *n* (%)	19 (24.7)	8 (25)	11 (24.4)	0.956
Smoking (yes), *n* (%)	6 (7.8)	2 (6.3)	4 (8.9)	0.511
Obesity (yes), *n* (%)	15 (19.5)	5 (15.6)	10 (22.2)	0.338
Living area, *n* (%)				0.353
Rural	29 (37.7)	14 (43.8)	15 (33.3)	
Urban	48 (62.3)	18 (56.3)	30 (66.7)	
**Associated comorbidities**				
Hypertension (yes), *n* (%)	55 (71.4)	22 (68.8)	33 (73.3)	0.855
IC (yes), *n* (%)	32 (41.6)	15 (46.9)	17 (37.8)	0.425
AFib (yes), *n* (%)	4 (5.2)	2 (6.3)	2 (4.4)	0.554
DM (yes), *n* (%)	7 (9.1)	2 (6.3)	5 (11.1)	0.379
CKD (yes), *n* (%)	4 (5.2)	2 (6.3)	2 (4.4)	0.554
Dyslipidaemia (yes), *n* (%)	6 (7.8)	1 (3.1)	5 (11.1)	0.199
CVI (yes), *n* (%)	7 (9.1)	4 (12.5)	3 (6.7)	0.313
**Time related factors**				
Time PA−SO (days), mean ± SD	1427.16 ± 1149.19	380.91 ± 575.29	2171.16 ± 828.08	**<0.0001**
LOS (days), mean ± SD	9.55 ± 4.06	10.41 ± 4.94	8.93 ± 3.22	0.274
**Preoperative laboratory analysis**				
Neutrophil count (×10^3/^µL), mean ± SD	5.95 ± 4.17	7.52 ± 6	4.83 ± 1.29	**0.022**
Lymphocyte count (×10^3^/µL), mean ± SD	1.75 ± 0.56	1.35 ± 0.44	2.03 ± 0.47	**<0.0001**
Monocyte count (×10^3^/µL), mean ± SD	0.61 ± 0.22	0.65 ± 0.18	0.58 ± 0.24	0.056
PLT count (×10^3^/µL), mean ± SD	264.06 ± 63.33	269.2 ± 67.1	260.4 ± 61.01	0.602
Glucose (mg/dL), mean ± SD	109.65 ± 27.91	109.41 ± 34.07	109.8 ± 22.96	0.954
APTT, mean ± SD	26.5 ± 4.03	26.79 ± 5.11	26.29 ± 3.08	0.596
INR, mean ± SD	2.25 ± 10.71	1.04 ± 0.13	3.12 ± 14.01	0.405
HCT (%), mean ± SD	41 ± 5.61	38.48 ± 6.9	42.79 ± 3.61	**0.004**
HGB (g/dL), mean ± SD	13.6 ± 1.95	12.83 ± 2.30	14.15 ± 1.44	**0.014**
CRP (mg/dL) mean ± SD	0.95 ± 0.33	1.08 ± 0.34	0.86 ± 0.3	**0.006**
**Serum Biomarkers**				
NLR (>2.63, cut-off), *n* (%)	43 (56.6)	27 (84.4)	16 (36.4)	**<0.0001**
PLR (>147, cut-off), *n* (%)	38 (49.4)	24 (75)	14 (31.1)	**<0.0001**
MLR (>0.31, cut-off), *n* (%)	44 (57.9)	26 (81.3)	18 (40.9)	**0.001**
SII (>605.31, cut-off), *n* (%)	45 (58.4)	29 (90.6)	16 (35.6)	**<0.0001**
SIRI (>83.34, cut-off), *n* (%)	39 (50.6)	26 (81.3)	13 (28.9)	**<0.0001**
AISI (>834.86, cut-off), *n* (%)	15 (19.5)	14 (43.8)	1 (2.2)	**<0.0001**

Abbreviations: PJI—periprosthetic joint infection; AF—aseptic failure; IC—ischemic cardiomyopathy; Afib—atrial fibrillation, DM—diabetes mellitus, CKD—chronic kidney disease; CVI—chronic venous insufficiency; Time PA−SO—time from primary arthroplasty to symptom onset; LOS—length of hospital stay; PLT—platelet; APTT—activated partial thromboplastin clotting time; INR − International normalized ratio (INR); HCT—hematocrit; HGB—hemoglobin levels; CRP—C−reactive protein; NLR—serum neutrophil lymphocyte ratio; PLR—serum platelet lymphocyte ratio; MLR—serum monocyte lymphocyte ratio; SII—serum systemic inflammation index; SIRI—serum systemic inflammation response index; AISI—serum aggregate inflammation systemic index. A *p* value of < 0.05 was accepted as being statistically significant (bold).

**Table 3:** Frequency of microorganisms identified in PJI group.

Microorganisms, *n* (%)	Frequency in All PJI Group (*n* = 32)
Methicillin-resistant *Staphylococcus aureus* (MRSA)	7 (21.88)
Methicillin-susceptible *Staphylococcus aureus* (MSSA)	10 (31.25)
*Streptococci*	3 (9.38)
*Klebsiella pneumoniae*	2 (6.25)
*Enterococcus faecalis*	1 (3.12)
*Providencia stuartii*	1 (3.12)
*Acinetobacter baumannii*	2 (6.25)
*Pseudomonas aeruginosa*	3 (9.38)
*Enterobacter cloacae*	1 (3.12)
Culture-negative	2 (6.25)

**Table 4:** Subgroup analysis between serum biomarkers and microorganism type.

Variables	Other Types of Microorganisms (*n = 13*)	*Staphylococcus aureus* (*n = 17*)	*p* Value
NLR (>2.63, cut-off), *n* (%)	9 (69.2)	16 (94.1)	0.134
PLR (>147, cut-off), *n* (%)	9 (69.2)	14 (82.4)	0.666
MLR (>0.31, cut-off), *n* (%)	10 (76.9)	14 (82.4)	0.531
SII (>605.31, cut-off), *n* (%)	10 (76.9)	17 (100.0)	0.070
SIRI (>83.34, cut-off), *n* (%)	10 (76.9)	15 (88.2)	0.628
AISI (>834.86, cut-off), *n* (%)	7 (53.8)	7 (41.2)	0.713

Abbreviations: NLR—serum neutrophil lymphocyte ratio; PLR—serum platelet lymphocyte ratio; MLR—serum monocyte lymphocyte ratio; SII—serum systemic inflammation index; SIRI—serum systemic inflammation response index; AISI—serum aggregate inflammation systemic index.

**Table 5:** Multivariate logistic regression of periprosthetic infection.

Variables	Periprosthetic Infection		*p* Value
	OR	95% CI	
NLR	17.52	4.65–66.66	0.006
PLR	9.45	3.08–29.39	0.021
MLR	6.25	2.14–18.28	0.028
SII	34.22	14.18–279.92	0.002
SIRI	6.64	2.39–18.40	0.024
AISI	10.66	3.56–31.95	0.011

Abbreviations: NLR—serum neutrophil lymphocyte ratio; PLR—serum platelet lymphocyte ratio; MLR—serum monocyte lymphocyte ratio; SII—serum systemic inflammation index; SIRI—serum systemic inflammation response index; AISI—serum aggregate inflammation systemic index; OR—odds ratio; CI—confidence interval. A *p* value of < 0.05 was admitted as being statistically significant (bold).

## Data Availability

The data used in this study can be requested from the corresponding author.
